# Acute cervical myelopathy with quadriparesis after cervical transforaminal epidural steroid injection: A case report

**DOI:** 10.1097/MD.0000000000018299

**Published:** 2019-12-16

**Authors:** Chunwoo Yang, Na Eun Kim, Jee Sun Beak, Na-Young Tae, Byeong Hun Eom, Byung-Gun Kim

**Affiliations:** Department of Anesthesiology and Pain Medicine, College of Medicine, Inha University, Incheon, South Korea.

**Keywords:** case report, complications, cord injury, epidural, injection, spinal cord

## Abstract

**Rationale:**

Cervical transforaminal epidural steroid injection (TFESI), can be an effective tool to improve pain associated with cervical radiculopathy. However, complications related to the procedure have been reported.

**Patient concerns:**

A 50-year-old woman who experienced acute cervical myelopathy with quadriparesis after cervical TFESI under fluoroscopic guidance.

**Diagnoses:**

The initial post-procedure cervical MRI revealed acute cervical myelopathy

**Interventions:**

She received 1000 mg of methylprednisolone was injected intravenously daily for 3 days

**Outcomes:**

Improvement in pain, with the only remaining complaints consisting of lingering mild pain in the left hand and occasional hypoesthesia

**Lessons:**

Cervical TFESI, despite careful fluoroscopic localization, resulted in spinal cord injury. A spinal cord injury may be treated with conservative treatments, such as medication and rehabilitation.

## 1 Introduction

Cervical radiculopathy is not a rare disease, with a prevalence of 83.2 cases per 100,000 individuals. It is commonly caused by compression of the cervical spinal nerve, which usually results from foraminal stenosis associated with cervical spondylosis (70%–75%) and herniated nucleus pulposus.^[1]^ With respect to treatment, a cervical transforaminal epidural steroid injection (TFESI) can be an effective tool to improve the pain associated with cervical radiculopathy.^[2]^ As conservative treatment for radiculopathy due to disc herniation and spinal stenosis in the cervical region, fluoroscopy-guided cervical TFESI is a useful technique to alleviate symptoms. TFESI has been performed in a large number of patients and its effects have been demonstrated in many studies.^[3]^ However, complications related to the procedure have been reported, including paralysis around the lips, tinnitus, vertigo, temporal limb paralysis, convulsions, cardiovascular toxicity, and even coma and death in serious cases. Studies suggest that more serious complications are caused by ischemia of the central nervous system due to vascular injury, vasospasm, and embolism associated with intravascular injections.^[4,5]^ Cervical TFESI requires special attention given the possibility of serious complications described in previous reports, including vertebral artery puncture despite close adherence to standardized procedures.

In the present report, we describe a case of spinal cord injury and left quadriparesis during a fluoroscopy-guided cervical TFESI for cervical spinal stenosis. We also discuss management options associated with this complication.

## 2 Consent

Written informed consent for treatment and publication of anonymized case details was obtained from the patient.

## 3 Case presentation

A 50-year-old woman visited the outpatient clinic of the authors’ hospital with complaints of radiating pain and a prickling feeling from the left cervical vertebra to the scapula and one arm that began 10 days before the visit. Her body weight, height, and body mass index were 62.1 kg, 151.3 cm, and 27.1 kg/m^2^, respectively; she had no history of trauma or underlying disease. Physical examination revealed that the neck pain worsened when the neck was tilted backward, and especially when extended to the left. The patient also complained of a bursting sensation of pain toward the left upper limb and tingling in the finger tips. There was no hypoesthesia, and the pain was distributed from vertebrae C4 to T1. Her numerical rating scale (NRS) score for pain (0 = no pain, 10 = most severe pain imaginable) was 7 to 8 points. Magnetic resonance imaging (MRI) revealed central canal stenosis at C3-4, C4-5, C5-6, and C6-7, and neural foraminal stenosis at C3-4, C4-5, C5-6, and C6-7. MRI results strongly suggested an indication for surgery. However, based on the absence of sensory or motor impairment and short symptom manifestation, the authors elected to use an epidural steroid injection technique and oral medication for treatment in the outpatient clinic with subsequent follow-up. Laboratory investigations performed before the procedure were normal.

**Figure 1:**
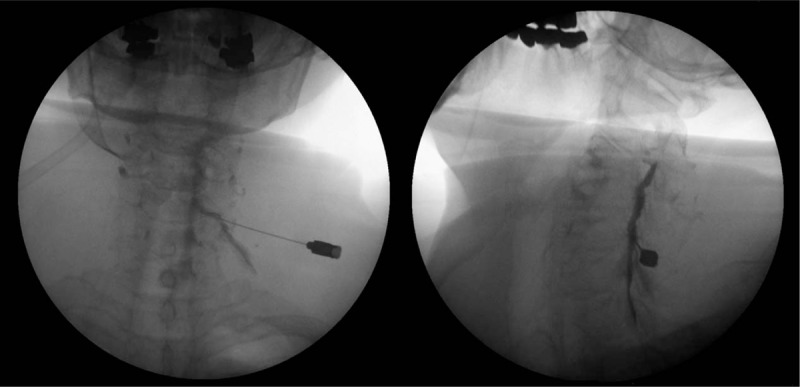
Fluoroscopy-guided left C6 cervical transforaminal epidural steroid injection. (A) Anterior-posterior view and (B) Oblique view showing proper needle location and spread of contrast media.

Fluoroscopy-guided cervical interlaminar epidural steroid injection was administered at the level of C7-T1 on her first visit, and then ≥2 injections were administered at an interval of one week. Although her NRS score decreased to 3 to 4 points, she still complained of a tingling sensation ranging from the left shoulder to the fingers. Thus, the authors decided to perform a left C6 cervical TFESI to deliver the drug to the anterior part of the epidural space and nerve root. The procedure was explained to the patient, and written informed consent was obtained. Electrocardiogram, oxygen saturation (SpO_2_), blood pressure, and consciousness of the patient were monitored throughout the procedure. The patient was placed supine on a table with the head slightly extended and turned away from the left side to be blocked. The overlying skin was prepared and draped in a sterile fashion, and 1% lidocaine was infiltrated at the needle insertion site. After the image intensifier (OEC series 9800, GE Heatlhcare, USA) was tilted and aligned perpendicular to the vertebral end plates in an anterior-posterior (AP) projection, it was rotated obliquely to the ipsilateral side by approximately 45° to provide the best view of the left C6 neural foramen. The procedure site was aseptically sterilized with chlorohexidine, followed by local anesthesia using 1% lidocaine. A 23-gauge spinal needle (B. Braun Medical, Germany) was carefully brought close to the anterior region of the facet joint with the assistance of the C-arm fluoroscope. As the tip of the needle touched the superior articular process, the block needle was moved approximately 1 mm forward to the front of the joint while keeping it adjacent to the bone. The depth of the needle was then verified in the AP view of the fluoroscope. The AP view ensured that the block needle was placed on the lower lateral side of the cervical pedicle and advanced no more than the medial one-third of the articular pillar. Once the ideal position of the block needle was determined, a total volume of 0.2 to 0.3 mL of contrast dye (Omnipaque, GE Healthcare, Dublin, Ireland) was injected under real-time fluoroscopic guidance to confirm needle placement and the absence of flow of the contrast agent into the vasculature (Fig. 1). After confirmation, a 4 ml mixture of 1% mepivacaine, dexamethasone 5 mg, and 1500 IU hyaluronidase was injected.

**Figure 2:**
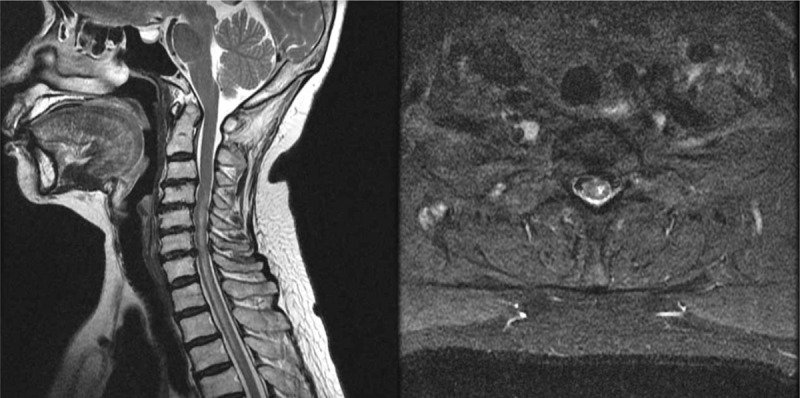
(A) Sagittal T2-weighted image of the cervical spine, intramedullary high signal intensity is seen at left sided spinal cord from C4 to T4 with ill-defined edema. (B) axial T2-weighted image of the cervical spine, showing intramedullary high signal intensity.

When approximately 1 ml of the medication was injected, the patient reported a shock-like pain radiating to the left hand. Considering she voiced no additional complaints of pain in response to inquiry, the authors believed that the pain was due to dural irritation. As the rest of the medication was injected, the patient complained of sudden pain in the posterior neck and the lateral part of the left upper limb, which spread to the fingers and lower limbs. The procedure was immediately discontinued and the block needle was removed. The patient reported hypoesthesia and motor weakness in the left upper limb. Approximately 10 minutes post-procedure, the patient noted weakness in the left arm and bilateral lower limbs, and temporarily lost consciousness. She was immediately placed on a bed and administered auxiliary breathing through an oxygen mask. Her heart rate was 100 beats/minute and blood pressure was 75/42 mmHg. After securing a venous route, 10 mg of ephedrine was administered intravenously along with fluid; blood pressure was restored and spontaneous breathing started. The authors initially believed the incident was a reaction to total spinal anesthesia with epidural puncture. However, paralysis of the upper left limb persisted, even after 4 hours when the patient had regained consciousness and the effect of the local anesthetic had dissipated. It was then decided to admit the patient for monitoring. Four to five hours after the procedure, her lower limb motor capacity had recovered to normal. However, hypoesthesia of the cortical sensory segment of C6 and the reduced motor capacity of the left shoulder joint and the finger muscles persisted. Physical examination during hospitalization revealed no abnormality in strength of the lower limbs. A strength test of the left upper arm revealed grade IV/V flexion in the left elbow and grade IV/V extension of left wrist, with grade III+/V extension of left elbow, abduction of the left fifth finger, and the pinching force of left fingers. The pinprick test revealed reduced tactile and vibrational sensations, and a 50% reduction in the left upper limb at C6 and lower levels. The pathological reflex test was positive for Hoffman's sign in the left upper limb, but no other pathological reflex was found. The results of the Spurling test, Lhermitte's sign, neck compression test, neck distraction test, and the shoulder abduction relief sign were negative. Thus, emergent cervical MRI was performed, which revealed intramedullary high-signal intensity at the left sided spinal cord from C4 to T4, with an ill-defined edema (Fig. 2). After consulting with the neurosurgeons, 1000 mg of methylprednisolone was injected intravenously daily for 3 days, but this injection was discontinued out of concern for adverse effects. The patient complained of sporadic bursting sensations of pain in the left upper limb and the posterior region of the neck. Pain management, including 150 mg pregabalin twice daily (Lyrica, Pfizer, Groton, CT), 75 mg extended-release tramadol HCl, and 650 mg acetaminophen fixed-combination tablets, was administered. The patient has been treated with continuous medication, rehabilitation therapy, and other conservative treatments.

**Figure 3:**
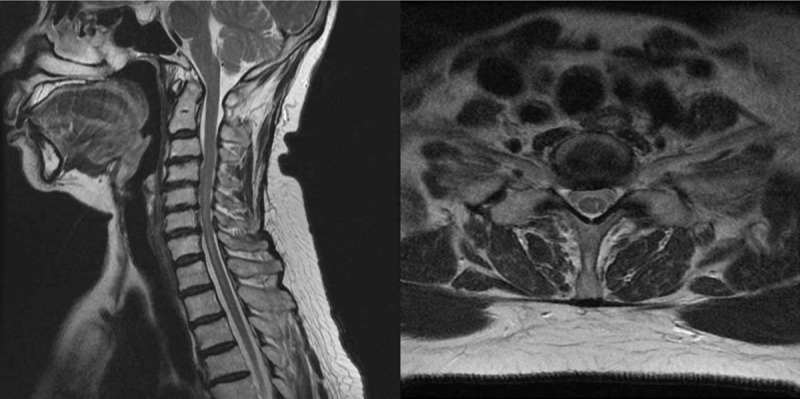
(A) Sagittal T2-weighted image of the cervical spine, the interval much decreased in the axial extent of the intramedullary high signal intensity at the left side spinal cord from C4 to T3, with decreased edema. (B) axial T2-weighted image of the cervical spine, showing decreased in the axial extent of the intramedullary high signal intensity.

At the 6-month follow-up, the patient's complaints consisted of only intermittent mild pain and hypoesthesia in the left upper limb, and mild dysfunction of fine motor skills in the left hand. Thus, left-sided stellate ganglion block was performed once per week, and medication, rehabilitation, and other conservative treatments were provided. Cervical MR images captured 7 months since the procedure revealed the interval much decreased in the axial extent of the intramedullary high signal intensity at the left side spinal cord from C4 to T3, with decreased edema (Fig. 3). In outpatient follow-up, the patient reported improvement in pain, with the only remaining complaints consisting of lingering mild pain in the left hand and occasional hypoesthesia. The patient recovered motor function sufficient to enable casual activity, but still experienced some difficulties with grasping, and first and second finger abduction due to reduced muscle force in the fingers. The patient is currently being monitored without particular treatment.

## 4 Discussion

Cervical TFESI is generally considered to be safe and is commonly recommended by physicians for the treatment of cervical radiculopathic pain.^[1]^ For radiculopathy due to cervical disc herniation and foraminal stenosis resistant to medication and other conservative treatments, the interlaminar approach with epidural steroid injection was the choice of treatment. Cervical TFESI was later introduced to gain close access to the lesion and the procedure has been guided using fluoroscopy. Cervical TFESI is useful in patients with pain from radiculopathy, and effective in symptom improvement with smaller amounts of drug compared with the amount required for epidural block based on the posterior interlaminar approach. This is because the drug can more easily reach the anterior part of the epidural space and the target nerve root. In recent years, a large number of cervical TFESIs have been performed, with reports of unanticipated serious complications, including limb paralysis and even death.^[6]^ There is also a case report describing complex regional pain syndrome type II after cervical TFESI.^[7]^

Despite the controversy regarding its safety, cervical TFESI has distinctive merit in conservative treatment. Risks for complication are always present with the procedure because it is exceedingly difficult to identify all major structures of the neck—even under fluoroscopic guidance—and the needle may invade the internal jugular vein, or carotid and vertebral arteries.^[8]^ There is also the possibility of serious complications including cerebral or spinal cord infarction, epidural hematoma, transient limb paralysis, and spinal cord injury. These complications have been reported since 2 cases of direct spinal cord injury in a C-arm-guided cervical epidural steroid injection were reported by Hodges in 1998.^[9]^ It was suspected that the spinal cord injuries in those cases occurred because the patients were sedated using anesthetics, including midazolam or propofol, and could not react to the needle touching the spinal cord. However, in contrast to the belief that patients would feel the painful sensation when the needle goes into the spinal cord, they do not because the brain and spinal cord do not have their own sensory nerves. It has been reported that even conscious patients do not feel pain during injection, and do not experience a tingling sensation or reduced motor function with a test dose of epidural anesthesia, even though the needle penetrates the spinal cord.^[10]^ Similarly, in our case, the patient did not experience a painful sensation when the cervical spinal cord was punctured, even though she was not sedated and was capable of communication. As a result, the medication was administered to the intramedullary space and caused acute spinal cord injury.

Spinal cord injury can be treated in the early stages using high-dose steroid therapy. An initial administration of 30 mg/kg of methylprednisolone slowly for ≥15 minutes, and additional administration of 5.4 mg/kg/hour of methylprednisolone for 24 hours, can prevent symptom deterioration and promote neurological recovery.^[11]^ However, in the present case, due to concerns for potential pulmonary complications^[12]^ and various other side effects of high-dose steroid injection, administration of steroid was limited. Judging from previous case reports and our own experiences, cervical TFESI requires preventive efforts because spinal cord injury is a complication with potentially serious consequences. Some have suggested the use of computed tomography (CT) guidance to avoid vital vessels^[13]^ and even argue to monitor the flow of contrast agent using digital subtraction angiography.^[14]^ However, spinal cord injury has also been reported to occur during CT-guided C7 TFESI,^[13]^ suggesting that it is not a completely safe method.

Some suggestions to minimize the risk for spinal cord injury during cervical TFESI have been offered. First, the correct needle position should be confirmed in the AP, lateral, and oblique planes using real-time fluoroscopy.^[15]^ Second, any complaint of lancinating pain during needle insertion should be considered a clear signal to immediately discontinue needle advancement.^[15]^ When the patient complains of upper limb pain or paralysis, physicians must consider the possibility of spinal cord penetration rather than radicular stimulation. Third, electrocardiogram, SpO_2_, blood pressure, and consciousness of the patient should be closely monitored throughout the procedure. Furthermore, it is better to perform the procedure on conscious patients.

In summary, we describe a serious complication of cervical TFESI, which, despite careful fluoroscopic localization and guidance, can result in spinal cord injury. A spinal cord injury may be treated with conservative treatment, including medication and rehabilitation therapy, in addition to natural healing for functional restoration. Moreover, they should be accompanied with continuous close monitoring of patient symptoms.

## Author contributions

**Conceptualization:** Byung-Gun Kim.

**Writing – original draft:** Chunwoo Yang, Jee Sun Beak.

**Writing – review & editing:** Byung-Gun Kim, Na Eun Kim, Byeong Hun Eom, Na-Young Tae.

**Conceptualization:** Byung-Gun Kim.

**Writing, original draft:** Chunwoo Yang, Na Eun Kim, Beak Jee Sun.

**Writing – review & editing:** Byung-Gun Kim, Na-Young Tae, Eom Byeong Hun.

Byung-Gun Kim orcid: 0000-0002-0036-0765.
